# NOVEL INSIGHTS ON THE RELATIVE IMPORTANCE OF CLINICAL AND GAIT MEASURES FOR DETECTING FALL RISK IN OLDER ADULTS

**DOI:** 10.1093/geroni/igz038.1771

**Published:** 2019-11-08

**Authors:** Sandra Hundza, Stuart MacDonald, Drew T Commandeur, Mark Klimstra

**Affiliations:** 1 University of Victoria, Victoria, British Columbia, Canada; 2 University of Victoria, Victoria, Canada

## Abstract

We sought to extend recent research that explored model-based approaches for combining clinical and gait measures to determine the most sensitive grouping for retrospectively classifying fallers from non-fallers which resulted in a model with 92% sensitivity and 66% specificity and an overall model of 83%. In the present study, the clinical assessment battery was augmented by incorporating more challenging balance items while removing clinical measures characterized by ceiling effects and restricted range. Thirty-two community-dwelling older adults (>70yrs, 16 fallers, 16 non-fallers) completed a battery comprising 76 measures of more challenging clinical measures of mobility and balance, and retained gait (GaitRITE), postural sway and physiological measures. Within each domain, highly collinear and theoretically-redundant measures were removed. Next, a Principal Component Analysis (PCA) identified those clinical and gait variables that accounted for the most unique variance. Finally, a backward stepwise logistic regression was performed on the reduced set of variables from the PCA to develop predictive equations. The current analysis yielded improved specificity of 75%, but slightly lower sensitivity 81%. Interestingly, when the results for the PCA from the previous study were used with the current data, the model classified fallers with 87% sensitivity and 86% specificity and an overall model of 86%. Notably, in all analyses, gait variables were central in identifying fall risk, with single- vs. dual-task difference scores of particular predictive importance. The differences observed between the best-fitting models across the two cohorts implies that modelling methods should accommodate and harness individual differences (e.g., machine learning techniques).

